# Development and implementation of a community health literacy hub, ‘Health Kiosk'—A grassroots innovation

**DOI:** 10.3389/fpubh.2022.1069255

**Published:** 2023-01-04

**Authors:** Caroline Masquillier, Kathleen Van Royen, Patricia Van Pelt, Dorien Onsea, Hilde Bastiaens

**Affiliations:** ^1^Department of Sociology, University of Antwerp, Antwerp, Belgium; ^2^Department of Family Medicine and Population Health, University of Antwerp, Antwerp, Belgium; ^3^Logo Antwerp, Antwerp, Belgium

**Keywords:** Health Kiosk, health literacy, healthy living, grassroots innovation, socioeconomically vulnerable groups, outreach working, integrated community care framework

## Abstract

Being health literate is important to get sufficient health information, to navigate the health system, to access appropriate care and to be able to self-manage health. As such it is a key determinant of health. There is a need for innovative measures to improve health literacy among people living in socioeconomically vulnerable circumstances. Literature shows that this innovation needs to: have “low-threshold access” to health resources in a community-based, outreaching way; be adapted to the needs of the target group; provide reliable and understandable health information adapted to the target population, and support people in developing confidence to act on that knowledge. In response to this need, this article describes—guided by the principles underpinning the Integrated Community Care (ICC) framework—the development and implementation process of a grassroots innovation, namely “Health Kiosk” in a socioeconomically vulnerable area in the northern part of a Belgian city. To be able to focus on the core activity of the Health Kiosk—i.e., stimulating healthy living and health literacy—community building and considering the spatial environment of the neighborhood formed a fundamental basis. Several core ingredients of the Health Kiosk are important to stimulate health literacy among socioeconomically vulnerable groups, namely: (1) working in a community-based, outreaching way; (2) providing accessible health information and support to act on that knowledge; and (3) working in a flexible and independent way to adapt to local needs. As such, the Health Kiosk forms a community health literacy hub with low-threshold access for people living in socioeconomically vulnerable circumstances.

## 1. Introduction

Decades of research underscore the strong relationship between socioeconomic position and health (1). People living in socioeconomic vulnerable circumstances are at higer risk of poor health than the general population (2). In addition, those who are often most in need of care, are amongst the least likely to receive it (2). Ensuring that no one is left behind in terms of health (1) is not only achievable through healthcare policy, but through an intersectoral approach addressing social determinants of health (3). Addressing these social determinants of health is fundamental to improving health and reducing long-standing inequities in health care (4).

Important in enabling people to overcome barriers to health and to improve social determinants of health is health literacy (5–8). Health literacy is a complex notion which entails competencies related to the process of accessing, understanding, appraising and applying health-related information. It influences health behavior and the use of health services (7). People with higher health literacy scores are more likely to get sufficient health information, are less likely to engage in risky behaviors and are more likely to report good self-rated health (5, 6). Evidence indicates that people with low health literacy engage more in risky health-behavior, have difficulty navigating the health system, have poorer health and higher mortality rates (5, 9). Thereby increasing health literacy may reduce health inequalities (10).

Literature shows that people living in socioeconomically vulnerable circumstances have lower levels of health literacy (5, 6). Authors conclude in a World Health Organization (WHO) report that “limited health literacy follows a social gradient and can further reinforce existing inequalities” [5: p. 19]. Targeted initiatives to improve health literacy can help address health inequities (5). However, a systematic review concludes there is “a lack of health literacy interventions for vulnerable social groups” [6: p. 54]. Therefore, innovative measures in real-world implementation research are needed to improve health literacy among people living in socioeconomically vulnerable circumstances and organizations working with them (5, 6, 9, 11).

Existing research regarding approaches targeting people living socioeconomically vulnerable circumstances, indicated that this innovation needs to adhere to the following principles. Firstly, community-based outreach initiatives are important, as research has shown that initiatives grounded in settings of everyday life are needed to reach the target group (5, 9, 12). As stated by Kickbush and colleagues, “communities are key settings for health literacy” [5: p. 40]. Daily health-related decisions are made in their homes and communities—usually acting as the primary source of health information (5). Secondly, the innovation needs to provide low-threshold access to health resources to ensure reaching the most excluded and to restore trust in the health system (6, 13). Besides providing reliable and understandable health information (5), it is important to support people in developing confidence to act on the required knowledge (5, 12), best achieved through more personal forms of communication (12). Interventions focusing on “capacity to act” [(14): p. 10] to improve a person's ability to gain access, understand and appropriately apply information are more likely to result in more effective health behavior (12). Thirdly, the innovation needs to be adapted to the needs of the target group, by being sensitive to and considering the diversity of the target population in terms of language, cultural, educational and socioeconomic characteristics (5, 6, 9).

This article describes the development and implementation process of a grassroots innovation, namely a community health literacy hub—“Health Kiosk”—aiming to improve health literacy for socioeconomically vulnerable populations.

## 2. Context

The Health Kiosk has been installed on a square in a socioeconomically vulnerable area in the northern part of a Belgian city. This area has a population of around 43,000 individuals in ~21,000 households, with a very high concentration of socioeconomically vulnerable groups (i.e., the target population of the Health Kiosk). The area is characterized by a high level of poverty, with more than half of newborns (58.5%) growing up in an economically vulnerable household. Almost half the population (47.7%) has right to increased compensation for healthcare costs. About three quarters (73.5%) of inhabitants has a migration background. In terms of healthcare usage, almost a third (32.1%) of inhabitants postponed a visit to a GP because they could not afford the care they needed (15). Local authorities requested the founder of the Health Kiosk to locate it on this square:

*The local policy makers have asked—because that was an explicit request—to set it on [name square], which is a square right next to the railway, one of the most difficult squares in [name city] with a high crime rate. So, we knew in advance that it was absolutely not an easy neighborhood, also because, there's quite a bit of drug use. But the explicit request was to set it up there, because something like a Health Kiosk, well, health could have a positive effect on the whole situation*.

*Founder of the Health Kiosk*.

## 3. Theoretical framework: Integrated community care framework

We will describe the Health Kiosk guided by the principles underpinning the Integrated Community Care (ICC) framework—designed by the Transnational Forum on Integrated Community Care (i.e., TransForm) (16). The ICC framework aims to improve quality of care and quality of life for individuals, families and communities by paying specific attention to “move beyond ‘delivery' of health and social care systems to genuine “co-development” with the individuals and communities that are traditionally seen as recipients” [16: p. 18]. The authors identify a typology based on three main dimensions distilled from collective reflection on real-world innovations: (1) main initiators and drivers; (2) center of focus; and (3) core ingredients. In addition, the ICC model proposes seven effectiveness principles to turn integrated community care aspirations into reality, as presented in Table 1.

**Table 1:** Effectiveness principles of the ICC framework [(16): p. 9–10].

**Number**	**Effectiveness principle**
**Co-develop health and wellbeing, enable participation**	
1	“Value and foster the capacities of all actors, including citizens, in the community to become change agents and to co-produce health and wellbeing. This requires the active involvement of all actors, with extra sensitivity to the most vulnerable ones.”
2	“Foster the creation of local alliances among all actors which are involved in the production of health and wellbeing in the community. Develop a shared vision and common goals. Actively strive for balanced power relations and mutual trust within these alliances.”
3	“Strengthen community-oriented primary care that stimulates people's capabilities to maintain health and/or to live in the community with complex chronic conditions. Take people's life goals as the starting point to define the desired outcomes of care and support.”
**Build resilient communities**	
4	“Improve the health of the population and reduce health disparities by addressing the social, economic and environmental determinants of health in the community and investing in prevention and health promotion.”
5	“Support healthy and inclusive communities by providing opportunities to bring people together and by investing in both social care and social infrastructure.”
6	“Develop the legal and financial conditions to enable the co-creation of care and support at community level.”
**Monitor, evaluate and adapt**	
7	“Evaluate continuously the quality of care and support the status of health and wellbeing in the community by using methods and indicators which are grounded within the foregoing principles and documented by participatory ‘community diagnosis' involving all stakeholders. Provide opportunities for joint learning. Adapt policies, services and activities in accordance with the evaluation outcomes.”

## 4. Programmatic elements

In what follows we will describe each of the three main dimensions of the Health Kiosk and its applicable effectiveness principles.

### 4.1. Main initiators and drivers

The Health Kiosk was founded by a grassroots initiator working at a local health network organization. Before applying for seed funding, the grassroots initiator contacted local organizations for collaboration, namely: a local college and a local university; local policy makers; a local school; a community health center; city sport services; local non-governmental organizations working around inclusiveness and solidarity; a community center and a local primary care network. These partners, which were mainly professionals and grassroots organizations, held several meetings during the start-up phase to further shape the concept of the Health Kiosk. During the start-up phase, the core steering group supported the initiator to create a support base in the community and to build trust among local organizations.

*I think that the period of creating support base was very important and the fact that we didn't set up the kiosk on the square immediately. I think it was a very important phase that we needed to go through. It also gave us the time to get to know the partners in the area, to sit down with them and to find out how they work, what the needs are and how they see things. We also want to take this into account when working with them, because they have been there longer than we have. They could also give us an idea of what the neighborhood is like and what we can expect*.

*Person running daily operations at the Health Kiosk*.

In line with ICC framework Principle 1 (see Table 1) (16), this core steering group has been critical not only for its support of the Health Kiosk in the start-up phase, but also during the daily operations for running the Health Kiosk: providing financial support; providing support for administrative and practical matters, such as financial administration; facilitating access to the public space; providing visual printed information materials, such as posters or leaflets on healthy eating and exercise; facilitating activities; providing advice; or organizing internships for students. Interns from the Bachelor in Social Work or from the Bachelor in Teacher Training for Physical Education and Exercise Recreation proved to be very important for the daily operations at the Health Kiosk by supporting the person managing the Health Kiosk. This person is essential for the daily operations at the Health Kiosk and helps bring all the organizations together, further embedding the project in the community and facilitating activities at the Health Kiosk. Those working at the Health Kiosk had to have a certain profile with specific personal traits and social skills, such as good networking skills, genuine interest in the target population, ability to build trust and give a warm welcome, and good organizational skills. The local community center was key to have access to electricity, toilets and Wi-Fi. Seed funding for the Health Kiosk was provided by a philanthropic organization, and later by local policymakers and a university research project. Regarding the latter, a collaboration with the European-funded SPICES research project from a local university facilitated documenting the activities of the Health Kiosk and gave credibility to the Health Kiosk as a new project.

### 4.2. Center of focus

To be able to focus on the core activity of the Health Kiosk—i.e., stimulating healthy living and health literacy—community building and considering the spatial environment of the neighborhood formed a fundamental basis.

#### 4.2.1. Community building

Building mutual trust in the community is essential and central to the success of the Health Kiosk in different ways—in line with ICC framework Principle 2 (see Table 1) (16). This trust basis was needed to embed the Health Kiosk in the community and to successfully reach local community members. Also by word of mouth did people from the community find their way to the Health Kiosk.

To embed the Health Kiosk in the community, building relationships with local community members and organizations who were respected in the neighborhood was crucial. For instance, at first the Health Kiosk was vandalized, but in collaboration with respected local community members this challenge was overcome. However, local organizations with a negative reputation could hinder the Health Kiosk embedding in the local community. For example, after the initial start-up phase, the initiators of the Health Kiosk realized that the community center, from where the initiators of the Health Kiosk started their activities, lacked trust from the community. This might have inhibited the initial integration into the community. Slowly trust was built by creating a presence on the square by distributing free fruit and soup; organizing small activities such as a partner day, a compliment day, a neighborhood day; a bench building workshop and many others. This slow social process to build trust is needed to become embedded in the community, but also for collaboration with local organizations:

*It's good that we have a partner who knows the neighborhood well, but you must be careful that it's a partner who has a positive [reputation]. This is a lesson to teach to others. It's something you don't know that from the start. It's really a matter of trial and error*.

*Person running daily operations at the Health Kiosk*.

The creation of local alliances between the organizations also facilitated collaboration among them to start-up health-related activities together. The person running daily operations at the Health Kiosk calls it “*connecting the dots*,” bringing partners together to organize activities where community members, local policy makers and members of the local organizations can participate. In addition, partner organizations started collaboration among themselves as well. Not all partners focus on health; some are more focused on welfare, youth, community, culture or employment. By collaborating with the Health Kiosk, health was brought to their attention as well—taking first steps toward improving health literacy among organizations.

*Alliances are also formed there, between those partners. They look for ways to work together, and we always try to add the health element to that. One of those goals is to increase resilience of that neighborhood and to reduce health inequities. […]Well, that's actually the ultimate goal in the long term. And the kiosk is like a lever to work across those sectors. That's actually the kind of discussions that we want to start*.

*Founder of the Health Kiosk*.

#### 4.2.2. Spatial environment

**Figure 1:**
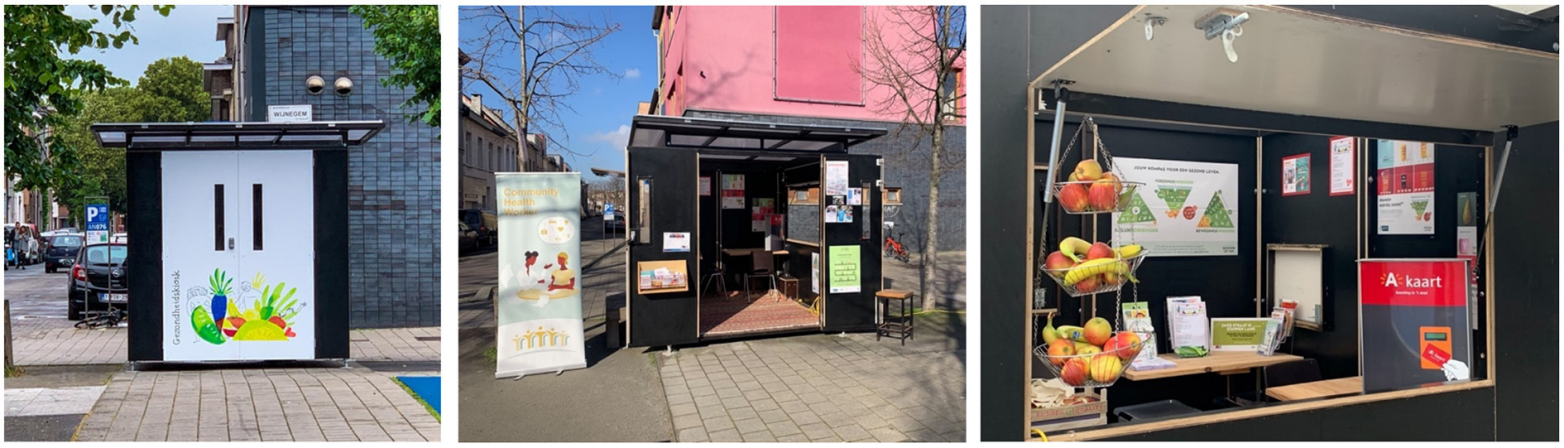
The Health Kiosk.

After the first steps were taken to build trust in the community, a physical Health Kiosk was placed in the public space. The central idea of the Health Kiosk is to work in an outreaching way, by being present in the public space with a small wooden structure that can be fully opened, without a door (see Figure 1). The open structure in a public space makes it approachable and stimulates community involvement. Additionally, a bench was installed next to the Health Kiosk that can be used for physical activity exercises. A local illustrator was invited to add illustrations to the doors showcasing health-related topics. These illustrations were also used for communication materials aimed at the local community.

#### 4.2.3. Care and wellbeing

The Health Kiosk's center of focus is related to the theme of care and wellbeing. In line with ICC framework Principle 4 (see Table 1) (16), the Health Kiosk aims to stimulate healthy living practices and health literacy among socioeconomically vulnerable groups. The focus is mainly on healthy living, but this can be understood in its broadest sense. Community members can discuss healthy eating and exercise, and join activities related to these topics. Additionally, attention is paid to mental health, healthy living space, dental health and parenting. Various health promotion activities were undertaken in collaboration with the core steering group as well as new partners who joined the project later. These will be further discussed in Section “Core ingredients”.

#### 4.2.4. Population reached

The Health Kiosk mainly reached people living in the community, both children and adults. The person managing the Health Kiosk summarizes that the Health Kiosk mainly reaches people “*who are vulnerable and who then live under the radar*.” In most cases, people live at a crossroads of different vulnerabilities: e.g., people living in poverty; people with a migration background; elderly people; people who have limited or no literacy; people living with a psychological vulnerability; people living on the streets; people who have a limited or no social network. Furthermore, because of its collaboration with local organizations, such as welfare organizations or organizations that work with newly-arrived migrants, the Health Kiosk also reached people living in socioeconomically vulnerable circumstances outside the community. Not specifically targeting certain vulnerable groups and leaving the door open to everyone who is curious or interested, contributed to reaching people:

*We must always make sure to establish a certain level of trust. I think you can do that much more easily in a smaller, simple environment, where people can come and go as they want*.

*Founder of the Health Kiosk*.

### 4.3. Core ingredients

Several core ingredients of the Health Kiosk are important to successfully stimulate health literacy among socioeconomically vulnerable groups: (1) working in a community-based, outreaching way; (2) providing accessible health information and support to act on that knowledge; and (3) working in a flexible and independent way to adapt to local needs.

#### 4.3.1. A community-based, outreaching way of working

The first core ingredient is that the Health Kiosk is an outreaching, low-threshold meeting place open to everyone. The open structure of the Health Kiosk (see Figure 1) in the middle of the square facilitates interaction. By working in an outreaching way, the aim is to reach people who live in socioeconomically vulnerable circumstances and empower them to take actions for their own health.

*Just standing there on the square without a door. All those shutters are open: it's just open. Otherwise you have to go to that door. Open that door. Go inside. Whereas now, we're just there. Very often there are questions like “What do you guys actually do? Who are you? Or what is this place?” Then you have a starting point and you can talk. The fact that there's free fruit, or that there's any fruit at all, that's also something different. I usually talk to people when they're just looking. It's different from sitting inside a building, which is much less accessible*.

*Person running daily operations at the Health Kiosk*.

First steps are taken to adhere to the ICC framework Principle 5 (see Table 1) [(16): p. 10]. The Health Kiosk forms a hub or meeting place connecting people and organizations in two ways: by referring people depending on their needs, or by providing space for organizations to work in an outreaching way by being present at the Health Kiosk.

*The kiosk is a binding agent. A binding agent between all those different partners in the neighborhood, but also a binding agent between the organizations and the neighborhood residents*.

*Founder of the Health Kiosk*.

#### 4.3.2. Accessible health information provision and support to act on that knowledge

The Health Kiosk facilitates low-threshold communication of evidence-based health information and supports people in putting this knowledge into practice.

##### 4.3.2.1. Low-threshold communication of evidence-based health information

Low-threshold communication of evidence-based health information was provided by making use of visual materials provided by other organizations, such as illustrated posters. For instance, a poster showing the number of sugar cubes in several types of drinks, such as soft drinks. Another example is the use of “Kamishibai,” a form of storytelling just using images. Exchange students created “Kamishibai” about healthy living and dental health, as part of their internship at the Health Kiosk. However, given the lack of easily understandable visual communication materials, it was challenging to inform to people with low literacy skills or who spoke a different language (a wide variety of languages are spoken in the community). To further overcome the language barrier, a collaboration was arranged with interns who speak languages common in the community. One of the interns at the Health Kiosk was a student in Social Work who fled Afghanistan. Due to her presence, the Afghan community was more engaged. If the people or interns running the Health Kiosk could not find a common language or communicated with people who have low literacy levels, body language or a translation app was used to inform people about healthy living. Moreover, the collaboration with a community health worker project further facilitated this low-threshold communication, given that they are members of the local community familiar with various languages.

*If you want to convey very important information to people, you have to translate it. You can do that through leaflets or through apps that allow people to hear the information in their own language. Many people are illiterate so they can't read: not even in their own language, nor are they able to write in their own language. That makes it very difficult, so if you can work with people from the communities and have them share the information, isn't that worth its weight in gold?*

*Founder of the Health Kiosk*.

A trust basis was essential to reach the target population. At first, people often visited just for a short conversation and to receive free produce. This then allowed further conversation, either during the same visit or during a next conversation, in which health information is provided and—if needed—referrals are made to health professionals present at the Health Kiosk or other local organizations. Others approached the Health Kiosk with a specific need or request for support or information.

*A very important point: if someone feel they can't trust you and the first conversation doesn't go well, they won't come again. They need to have a good feeling about you in order to keep coming. If they don't, then not many people will come to the kiosk and it won't work*.

*Intern at the Health Kiosk*.

##### 4.3.2.2. Facilitation to put health information into practice

Besides providing information, an important element of the Health Kiosk is helping people put this information into practice. This was achieved by distributing free produce and by providing free access to healthcare professionals. First, free produce was distributed, which often served as a first contact point with community members. As a result of a collaboration with a local wholesaler, the Health Kiosk receives free fresh produce weekly to distribute for free.

*It's actually a meeting place where that people can get information around health in a very accessible way. It's very simple. A place where they can come to ask questions, where they can get a piece of fruit, where they are welcome*.

*Founder of the Health Kiosk*.

Second, first steps were taken to provide people with guidance along the healthcare continuum—from prevention to care. Each of the activities organized for this goal were adjusted to suit the needs of people living in socioeconomically vulnerable circumstances. Community members were invited to free screening days—in collaboration with medical students from a local university—where they received free measurements of the following parameters: blood pressure, abdominal girth, fat percentage and Body Mass Index, glycemic index, cholesterol, resting heart rate and post-exercise heart rate. These measurements form the basis for receiving advice on eating and exercise and/or further referral. For instance, people can be referred to a free dietician and movement coach who come to the Health Kiosk every month, or to the community health workers who can facilitate access to affordable healthcare.

*The kiosk is a meeting place. At the same time, it's a place from which we organize a number of things, such as the medical students who will now come every month to measure parameters, a dietician who will be present every month from February, a movement coach who will be present every month. […] The community health workers who have their base there visit once or twice a week. It's just a meeting place for people, for organizations, but also a place where you can find information or ask questions or seek help for problems, for someone in pain, for all kinds of things*.

*Founder of the Health Kiosk*.

#### 4.3.3. Flexible and independent way of working to adapt to local needs

To establish the Health Kiosk, a lot of flexibility was required from the team members, funders and partner organizations. As such, it became a space for creative experimenting to respond to local needs by taking the time to listen and observe the community. For instance, in the beginning information sessions about healthy eating were organized but few or no people showed up—this taught the organizers that the same message needed to be conveyed in other ways.

*Now I see it more as an experimentation place or a lab where all kinds of things can develop and grow, where ideas of people come together and lead to something*.

*Founder of the Health Kiosk*.

The Health Kiosk's independence—i.e., being unrelated to any specific organization—has allowed it to be very flexible and to develop its organization and activities bottom-up, and made it easy to adapt to local needs of community members. Collaborating with organizations from different backgrounds provided different types of expertise. However, this independence also presented practical challenges: there is no policy-level regulatory framework in which the Health Kiosk could operate and finding funding to sustain the Health Kiosk was difficult, among others.

## 5. Discussion

This paper described the development and implementation process of a community health information hub with “low-threshold access” for people living in socioeconomically vulnerable circumstances. To be able to focus on the core activity of the Health Kiosk—i.e., stimulating healthy living and health literacy—community building and considering the spatial environment of the neighborhood formed a fundamental basis. Several core ingredients of the Health Kiosk are important to successfully stimulate health literacy among socioeconomically vulnerable groups, namely: (1) working in a community-based, outreaching way; (2) providing accessible health information and support to act on that knowledge; and (3) working in a flexible and independent way to adapt to local needs.

The Health Kiosk is an example of a grassroots innovation, which is “a network of activists and organizations generating novel bottom–up solutions for sustainable development and sustainable consumption; solutions that respond to the local situation and the interests and values of the communities involved” [(17): p. 1]. The concept of the Health Kiosk evolved by experimenting in the field and by collaborating with local organizations. It was a space for experimentation to discover what works for whom and which strategies should not be continued. Flexibility was required from the organizers, but also from the funders to allow for adaptation along the way. After the philanthropic and research funding ended, it proved to be challenging to find sustainable funding. This is in line with previous research on community-based care initiatives that stated that “governments and development groups may favor investment in interventions with easily measurable indicators and may underinvest in the more intangible social processes and community participation that are critical to longer-term success and sustainability” [(18): p. 8].

The analysis of the Health Kiosk was guided by the principles underpinning the ICC framework (16); Principles 1, 2, 4 and 5 (see Table 1) were applicable to some extent. In line with Principle 2, the set-up of the Health Kiosk was similar to other community-based care programs: a “slower social process […] can be required for establishing stronger ties” with the community [(18): p. 8]. In this process, it is important to collaborate with organizations who are already embedded and trusted by the community—a finding in line with the implementation of other outreach programs (19). Furthermore, it is important to have a dedicated person facilitating this slow social process of embedding the Health Kiosk in the community, and who manages the Health Kiosk. This person is important in setting up the Health Kiosk and engaging all potential partners in the neighborhood at different levels—ranging from the socioeconomically vulnerable groups, to welfare and health actors, and local policy makers. However, unlike Principle 1 articulates (16), community members themselves were not actively involved in the decision-making processes of the Health Kiosk. Future development of the Health Kiosk could focus on collaborating with volunteers who share a lived experience with members of the target population. These volunteers understand local knowledge and attitudes about health (20) and have a unique understanding of the experiences, language, culture and socioeconomic realities of the people they support (21, 22). Proper guidance and follow-up for these volunteers is essential, as the conversations held in the Health Kiosk can be emotionally taxing. By involving the socioeconomically vulnerable groups in the development of the Health Kiosk, this project could become a grassroots innovation that is also inclusive. Recently, scholars have argued that “inclusive innovation provides an avenue to address challenges related to poverty, inequality and exclusion by bringing people and organizations who were out of the spotlight, to the mainstream development activity” [(23): p. 2].

ICC framework Principles 3, 6 and 7 were not yet applicable to the Health Kiosk (see Table 1) (16), but these principles provide avenues for future aspirations. In line with Principle 3, it would be very valuable if the Health Kiosk could focus on strategies that integrate people's life goals into its activities to stimulate motivation and encourage long-term behavior changes (24, 25). Furthermore, closer collaboration with primary healthcare actors forms an additional future focus point, so that people can easily be referred from the Health Kiosk and vice versa. In line with Principle 6, co-creation with members of the neighborhood and involving them in the decision-making processes of the Health Kiosk's activities and vision are important focus points—aiming to create more community ownership. Moreover, it would be interesting to investigate whether the workings of the physical Health Kiosk could be supported by technological innovations, such as a referral platform to health and welfare organizations—as done at a “Gesundheitskiosk” in Germany (26). First steps were taken to adhere to ICC framework Principle 7 (see Table 1) (16). The workings of the Health Kiosk were adapted along the way by learning from experiences in the field—in line with the ICC's vision on measuring progress, maintaining a longitudinal view and vision to leave room for “the inevitable but very necessary learning curve” [(16): p. 11].

## 6. Conceptual and methodological constraints

An important limitation of this paper is the methodological constraints. Only a limited number of in-depth interviews was conducted—after obtaining written informed consent. The following key actors of the Health Kiosk participated in an in-depth interview: the founder of the Health Kiosk; the person running the daily operations; and an intern. The interviews were audiotaped, which allowed us to produce detailed interview transcripts. The transcripts were imported into NVivo (version 1.5) for thematic analysis. Data was carefully analyzed by reading and re-reading the field notes and transcripts of interviews. First, the data was open coded. In this phase of data analysis, primary information categories which remain close to the original data were constructed. These open codes were then categorized in the axial coding phase to identify patterns and regularities emerging from the data. The categories which emerged from the axial coding were integrated in the subsequent phase of selective coding. Concepts were systematically refined as the data were collected and analyzed. In this process, specific attention was paid to remaining close to the gathered data (27). These findings were additionally complemented by documents, such as meeting notes. Even though considered as a potential bias, the close involvement of the research team in all the steps of the development of the Health Kiosk allowed them to document the development and implementation process from a participatory point of view. At the time of the in-depth interviews, the researcher, who conducted the interviews and analysis had not been involved in the development of the Health Kiosk, providing her with a more independent role as a researcher. In addition, respondent validation was conducted by returning the synthesized analyzed results to the respondents to verify accuracy and responance with their experiences (28, 29).

This important methodological limitation also reflects the challenges of monitoring and evaluating this initiative (see ICC framework Principle 7, Table 1) (16). As part of a larger research project, which provided financial and substantive support to the Health Kiosk, a diary study was initiated. In this study, the person managing the Health Kiosk and local organizations present at the Health Kiosk were invited to fill in a diary. However, this research method proved to be difficult to complete by the members of the Health Kiosk during their day-to-day work, causing this research method to be stopped. Currently, a “city loyalty card scanner” is present in the Health Kiosk, where people can scan their city cards, with which they can receive access to various free activities (e.g., a ticket for the swimming pool). At the same time, this “city loyalty card stand” anonymously measures who visits the Health Kiosk. A future avenue for research could be finding innovative ways to monitor the activity at the Health Kiosk, also integrating the vision of those reached and those involved in the daily operations of the Health Kiosk. Methods, such as, participatory research methods with socioeconomically vulnerable community members and participatory observation could serve as a starting point for the evaluation (16).

Another limitation of the Health Kiosk is that it currently does not specifically address different health literacy competencies (7). For future iterations, it is important to focus on stimulating the “competencies related to the process of accessing, understanding, appraising and applying health-related information” [(7): p. 9]. Furthermore, future research could explore whether health information and practices are shared within families and households after a person visits the Health Kiosk for produce or a conversation about health. Moreover, research is needed to investigate the way in which the Health Kiosk can be aligned more with the health system.

Despite its current limitations, the Health Kiosk forms a promising innovation to improve health literacy among people living in socioeconomically vulnerable circumstances and organizations working with them—both in high-income countries, such as the setting of this paper, as well as in low-and middle-income countries. The Health Kiosk is a grassroots innovation that responds to the need recently identified in a systematic literature review that “intervention programmes for minority populations with low rates of literacy are scarce and little known” [(6): p. 61].

## Data availability statement

The datasets presented in this article are not readily available because protection of the respondents' privacy. Requests to access the datasets should be directed to caroline.masquillier@uantwerpen.be.

## Ethics statement

The studies involving human participants were reviewed and approved by the Ethics Committee of Antwerp University Hospital (B300201940009). The patients/participants provided their written informed consent to participate in this study.

## Author contributions

Grassroots innovators: PV and DO. Methodology: CM, KV, and HB. Formal analysis, investigation, and writing—original draft preparation: CM. Funding acquisition: HB. Writing—review and editing: all authors.
